# TDP-43 Regulates Drosophila Neuromuscular Junctions Growth by Modulating Futsch/MAP1B Levels and Synaptic Microtubules Organization

**DOI:** 10.1371/journal.pone.0017808

**Published:** 2011-03-11

**Authors:** Vinay K. Godena, Giulia Romano, Maurizio Romano, Chiara Appocher, Raffaella Klima, Emanuele Buratti, Francisco E. Baralle, Fabian Feiguin

**Affiliations:** 1 International Center for Genetic Engineering and Biotechnology, Trieste, Italy; 2 Department of Life Sciences, University of Trieste, Trieste, Italy; Skirball Institute of Biomolecular Medicine - New York University Medical Center, United States of America

## Abstract

TDP-43 is an evolutionarily conserved RNA binding protein recently associated with the pathogenesis of different neurological diseases. At the moment, neither its physiological role *in vivo* nor the mechanisms that may lead to neurodegeneration are well known. Previously, we have shown that TDP-43 mutant flies presented locomotive alterations and structural defects at the neuromuscular junctions. We have now investigated the functional mechanism leading to these phenotypes by screening several factors known to be important for synaptic growth or bouton formation. As a result we found that alterations in the organization of synaptic microtubules correlate with reduced protein levels in the microtubule associated protein *futsch*/MAP1B. Moreover, we observed that TDP-43 physically interacts with *futsch* mRNA and that its RNA binding capacity is required to prevent *futsch* down regulation and synaptic defects.

## Introduction

TDP-43 is an RNA binding protein of 43 kDa that belongs to the hnRNP family and plays numerous roles in mRNA metabolism such us transcription, pre-mRNA splicing, mRNA stability, microRNA biogenesis, transport and translation [1], [2]. TDP-43 is very well conserved during the evolution, especially with regards to the two RNA-recognition motifs (RRMs), the first (RRM1) being responsible for the binding of TDP-43 with UG rich RNA [3]. In consonance with these described functions, TDP-43 prevalently resides in the cell nucleus where it co-localizes with other members of the RNA processing machinery [4]. Nevertheless, in pathological conditions such as amyotrophic lateral sclerosis (ALS) and frontotemporal lobar degeneration (FTLD), TDP-43 appears in the form of large insoluble protein aggregates redistributed within the cytoplasm [5]. At the moment, however, it is not clear how these alterations may lead to neurodegeneration. In theory, the cytosolic accumulation of TDP-43 may induce a toxic, gain of function effect on motoneurons whilst the exclusion of TDP-43 from the cell nucleus may lead to neurodegeneration due to a loss of function mechanism. At present, several lines of evidence mainly from different cellular and animal models support either view suggesting that both models may be acting to lead the disease condition [6], [7]. Recently, to determine the physiological role of TDP-43 *in vivo* we have reported that the flies which lack the TDP-43 homologue (TBPH) closely reproduce many of the phenotypes observed in ALS patients, such as progressive defects in the animal locomotion and reduced life span [8]. Moreover, we have observed that loss of TDP-43 function in *Drosophila* resulted in reduced number of motoneurons terminal branches and synaptic boutons at neuromuscular junctions (NMJs), indicating TDP-43 may regulate the assembly and organization of these structures. In coincidence with that, it should be noted that overexpression of TDP-43 in Drosophila has been reported to increase dendritic branching [9], lead to motor dysfunction and reduced life span [10], axon loss and neuronal death [11], is generally toxic regardless of inclusion formations [12], and at least in part is the cause behind the degeneration associated with TER94 mutations which is the Drosophila homologue of the VCP protein [13]. Taken together, and in consideration that Drosophila TDP-43 (TBPH) can functionally substitute for human TDP-43 in functional splicing assays [14], all these reports confirm that Drosophila may represent a highly suitable animal model to investigate TDP-43 functions both in normal and disease conditions.

Drosophila larval NMJ is a well-characterized system to analyze the cellular and molecular events that are involved in synapse development and plasticity [15]. Synaptic growth during larval development is expanded according to muscle size and is accomplished by the addition of new boutons to the existing presynaptic terminals [16]. Typically, defects in synapse formation and synaptic growth are linked to cytoskeleton abnormalities, since the synaptic boutons and the newly formed buds require the underlying presynaptic microtubules to maintain their structural organization and plasticity inside the innervated muscles. Thus, to determine the physiological role of TDP-43 *in vivo* and the pathological consequences of its altered function, we decided to analyze in depth the molecular organization of *Drosophila* NMJs during larval development in TDP-43 minus flies.

## Results

### TDP-43 is Required for Synaptic Growth and Bouton Shape

**Figure 1:**
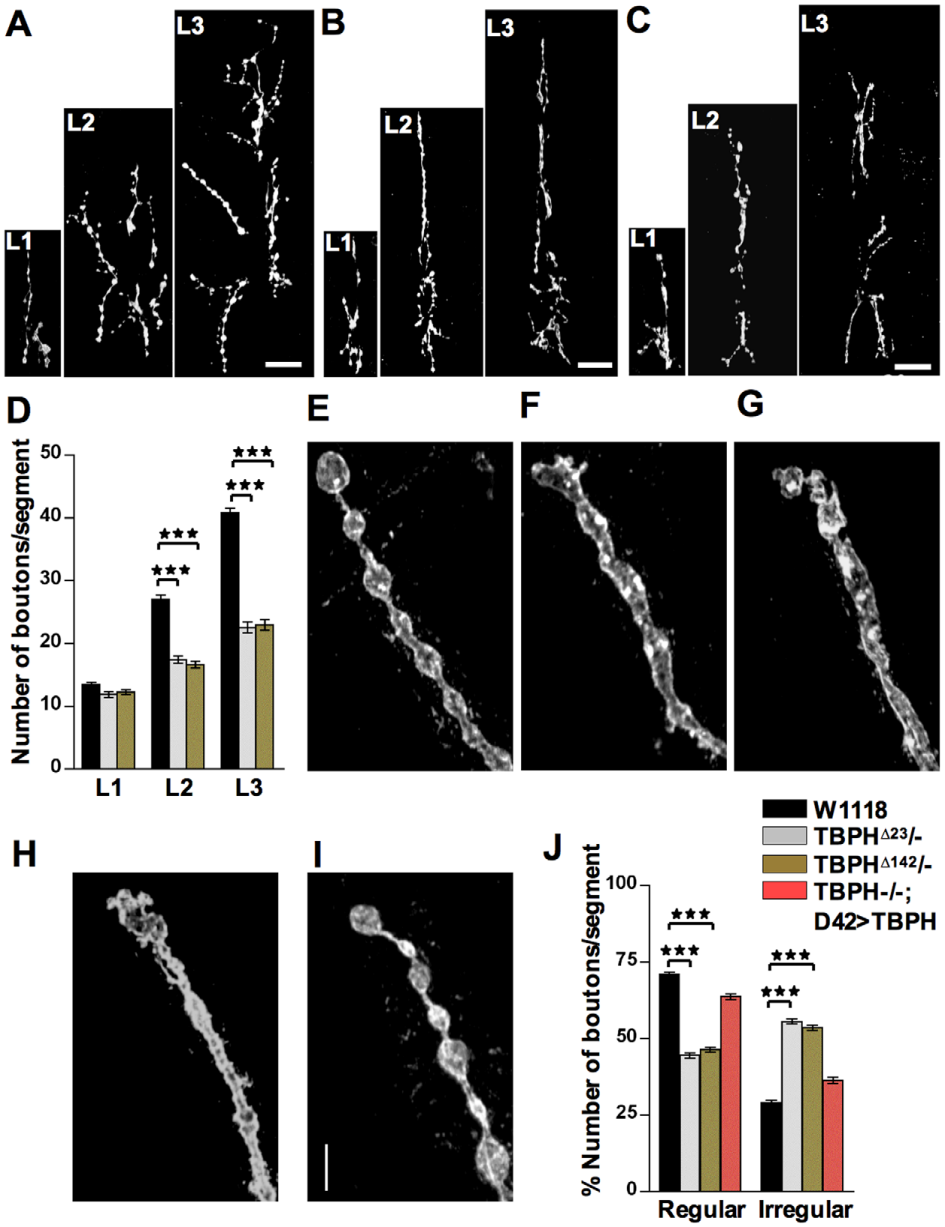
Loss of TBPH function affects synaptic growth and boutons shape. (A) Confocal images of wild type NMJs on muscle 6 and 7, abdominal segment II, at L1 (38 h), L2 (62 h) and L3 (110 h) stages using α-HRP antidody. (B) And (C) Similar α-HRP staining showing NMJ morphology at different stages of larval development in TBPH^D23^ and TBPH^D142^ homozygous larvae. Scale bar 20 µm. (D) Quantifications showing total number of boutons present in the abdominal segment II of wild type and TBPH mutant alleles during larval development. (E) Regular shape and distribution of 1b boutons in wild type NMJs compared to misshapen boutons in (F) TBPH^D23^/- (G) TBPH^D142^/- and (H) TBPH^D23^/+;*elav*>TBPH-RNAi mutants. (I) Bouton shape is rescued by expressing UAS TBPH in motor neurons with *D42*-GAL4. Scale bar 5 µm. (J) Quantifications showing regular and irregular boutons present in the abdominal segment II at third instar larval stages in wild type and mutant alleles. *** Indicates p<0.001 calculated by one-way ANOVA. Error bars indicate SEM.

Growth and formation of motoneurons synaptic terminals at the neuromuscular junctions (NMJs) in *Drosophila melanogaster* entails continuous addition and stabilization of new synaptic boutons to accommodate the rapidly growing larval muscles during development [17]. It was recently described also by us and other researchers that loss of function mutations in TDP-43 induced locomotive defects and influenced the morphological organization of the NMJ [8], [18]. However the mechanisms behind these phenotypes, whether they were due to defects in synaptic growth or stabilization, are not known. To explore these possibilities, we now decided to quantify motoneurons terminal branches formation and expansion, together with the number of big synaptic boutons (1b) created on muscles 6 and 7 at different stages of larval development. For these experiments we analyzed NMJs organization from 38 hrs first instar larvae (L1), 62 hrs second instar (L2), to matured 110 hrs third instar larvae (L3), using two different TBPH mutant alleles. The neuronal membrane marker anti-HRP was used to determine whether synaptic terminal defects occur in TBPH minus alleles. No significant differences in the NMJ morphology during first instar larval stages (L1) were detected in two TBPH minus alleles compared to wild type controls (Figure 1A–C L1 and 1D for quantifications, wt = 13.4±0.4 boutons, TBPH^D23^ = 11.8±0.46 boutons, TBPH^D142^ = 12.2±0.38 boutons, *n* =  16 larvae, *p*>0.05. We also observed that presynaptic ramifications and muscular insertions appeared normal in L1, TBPH mutant motor axons. In contrast to this, we observed significant abnormalities in L2 TBPH mutants regarding to NMJ morphology (Figure 1A–C L2 and 1D, wt = 27±0.67 boutons, TBPH^D23^ = 17.4±0.58 boutons, TBPH^D142^ = 16.6±0.53 boutons, *n* =  16 larvae, *p*<0.001). The synaptic terminal defects were even more evident in third instar larval stage (L3), where a very little addition of new synaptic boutons was detected in TBPH mutant flies (Figure 1A–C L3 and 1D, wt = 41±0.80 boutons, TBPH^D23^ = 22.5±0.87 boutons, TBPH^D142^ = 23±0.87 boutons, *n* =  17 larvae, *p*<0.001). Muscle development was normal in TBPH mutant flies since no differences in organ growth, cytoskeleton organization or postsynaptic differentiation were observed regarding to wild type controls (Figure S1A–F and G). Thus, these experiments indicated that TBPH function in motoneurons promotes presynaptic growth and the addition of new synaptic boutons during larval development.

In addition, we analyzed whether synaptic stability was compromised in TBPH minus larvae. For these experiments, we used a previously established assay to quantify presynaptic retraction events in NMJ. This assay is based on the evidence that formation of postsynaptic structures in *Drosophila* muscles depends on the presence of the pre-synaptic terminals [19]. Therefore, postsynaptic resident proteins will only be present at sites where presynaptic terminals are located. Consequently, postsynaptic sites that do not have opposite presynaptic neuronal markers, identify regions of the NMJs where the nerve terminals once were present and retracted leaving their “footprints” [20]. To quantify synaptic retraction events or footprints we used a postsynaptic marker protein Discs-large (Dlg) and anti-HRP staining to label presynaptic terminals [21]. Double-labeled NMJ of third instar larvae were analyzed and no significant differences in the number of footprints were found in TBPH minus flies (Figure S1 Hiv-Hvi TBPH^D23^, Hvii–Hix TBPH^D142^) compared to wild type controls (Figure S1 Hi–Hiii and I for quantifications). These data further suggested that the NMJ defects observed in TBPH mutants were due to a lack of new synaptic bouton formation rather than to increased presynaptic retraction. In order to control that the TBPH loss of function defects at motoneuron synaptic terminals were not due to a more general problem of neuronal degeneration, we determined the viability of these motoneurons by expressing the GFP protein using the *D42*-GAL4 driver in both wild type and TBPH mutant flies. Larval brains were dissected at third instar stage and stained with the neuronal marker *elav* (Figure S2A). The number of GFP positive neurons in the terminal abdominal segment, in particular a4-a5-a6-a7, of the dorsal cluster of the ventral nerve cord was counted [22]. We found that the viability of motoneurons was not affected in TBPH mutant flies (Figure S2B) indicating that the synaptic phenotypes described above were specific for TBPH function.

Defects in synaptic growth as described in TBPH mutants were very often associated with alterations in synaptic bouton shape. We therefore analyzed the morphology of individual big synaptic boutons present on muscles 6 and 7 in L3 wild type and TBPH mutant larvae. Staining with anti-HRP antibodies showed that, wild type 1b boutons were rounded and had a smooth surface with a uniform distribution along the presynaptic terminals that resemble the “beads on a string” (Fig. 1E) [23]. TBPH^D23^ and TBPH^D142^ mutant synaptic boutons appeared deformed and were irregularly spaced along the terminal axons with several fused or elongated silhouettes and clear loss of their characteristic round-smooth shape (Figure 1F–G). Similar alterations were observed in TBPH-RNAi expression in neurons by using the *elav*-GAL4 driver (Figure 1H), implying the neuronal origin of these defects. Although the analysis was centered on muscles 6 and 7, aberrant boutons were detected in almost every body muscle examined. This bouton phenotype was highly penetrant and specific for TBPH gene function since genetic rescues performed in TBPH mutant neurons, obtained by expressing the endogenous TBPH protein with *D42*-GAL4 driver, was able to recover the phenotype (Figure 1I, and 1J for quantifications). Thus, these experiments demonstrated that TBPH function is required in motoneurons to sustain synaptic growth and boutons shape.

### Testing for Potential Alterations in Cytoskeletal Factors that Sustain Synaptic growth and Bouton shape

The exponential growth of *Drosophila* NMJ during development occurs by the addition of new boutons. Newly formed boutons originate after budding from their parent boutons and NMJ expansion takes place by extending neural processes and bouton enlargement. This entire process is mainly supported by the underlying presynaptic cytoskeleton and induced us to hypothesize that alterations in the organization of these structures might explain the morphological defects observed in TBPH mutant alleles. In support of this idea, it was previously described that NMJ defects similar to TBPH loss of function were found in several other mutants that induce alterations in the organization of the microtubules (MT) at the synaptic terminals [24], [25].

**Figure 2:**
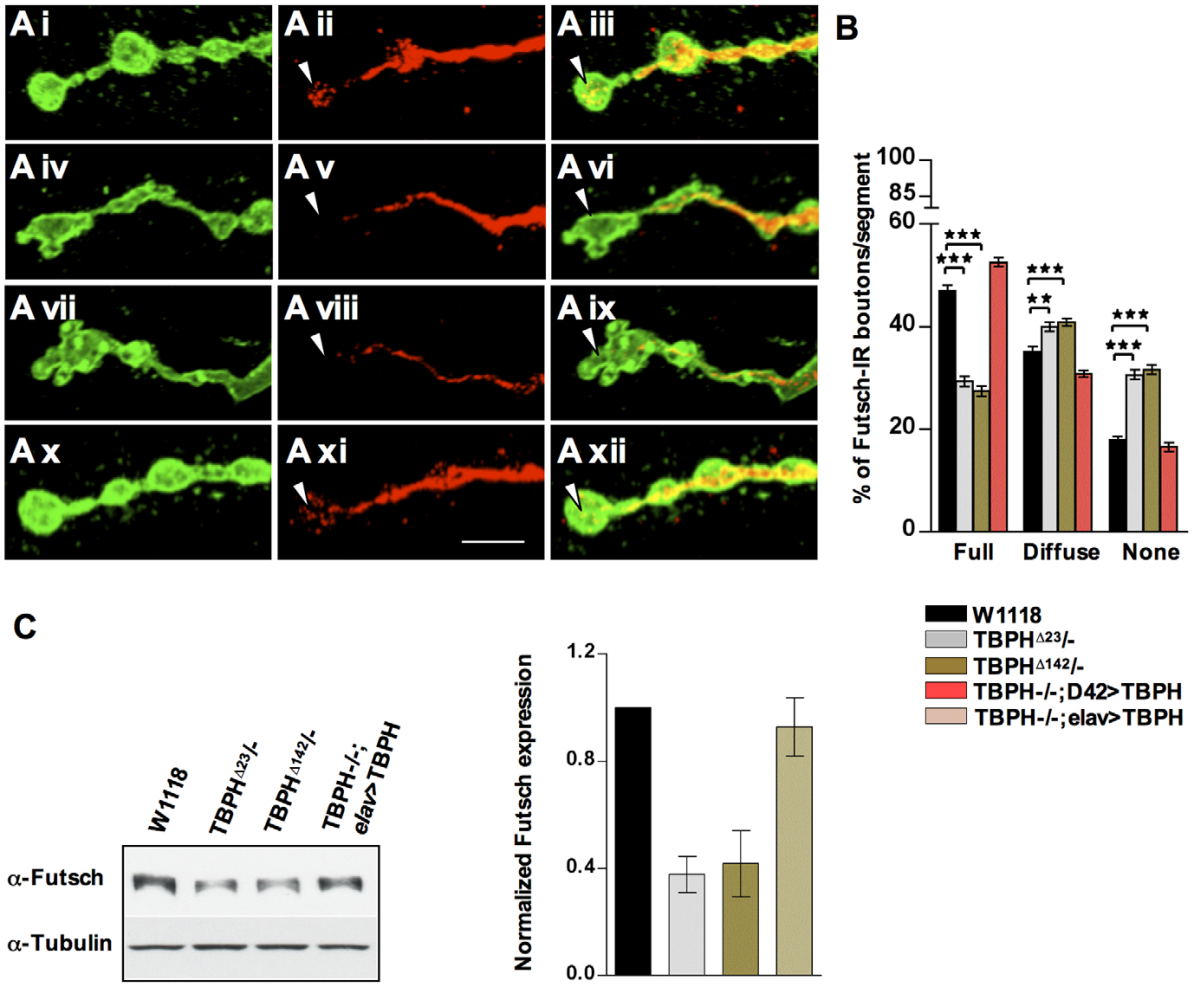
Loss of TBPH interferes with microtubule organization. (A) Confocal images showing *futsch* staining at the terminal synaptic boutons of (Ai–Aiii) wild type, (Aiv–Avi) TBPH^D23^, (Avii–Aix) TBPH^D142^. Note the complete absence of *futsch* at the most distal, newly formed, boutons in TBPH mutants (arrow head). (Ax–Axii) Expression of TBPH protein by *D42*-Gal4 rescues *futsch* staining in TBPH mutant larvae. (B) Quantifications of *futsch* staining pattern in muscle 6–7 abdominal segment II showing increased number of diffused *futsch* and *futsch* negative boutons in TBPH mutant alleles compared to wild type. *n* = 15 larvae. **p<0.01 and ***p<0.001 calculated by one-way ANOVA. (C) Western blot analysis and the respective histogram confirmed the reduced *futsch* expression levels in TBPH mutant fly heads compared to wild type. *n* = 4.

Based on these considerations we therefore decided to test for alterations at the level of important factors known to play a role in synaptic organization at the cytoskeletal level. Regarding to that, the intracellular localization of different presynaptic proteins involved in synaptic function such as Bruchpilot [26] and Synapsin [27] were not affected by the absence of TBPH function (Figure S3A,B). Similarly, we did not find alterations in the expression levels of the adhesion protein Fasciclin II (Figure S3C) [28], type II BMP receptor Wit-C (Figure S3D) [29], Bruchpilot (Figure S3E) and the cytoskeletal protein Spectrin (Figure S3F) [30]. For the contrary, our experiments detected that the protein levels of *futsch* were consistently reduced in TBPH minus heads (Fig. 2C). This was considered particularly interesting since *futsch* is a neuron-specific microtubule binding protein homolog to human MAP1B responsible for maintaining MT integrity at presynaptic terminals during NMJ expansion [31]. In addition, mutations in *Drosophila* MT binding protein *futsch* reproduce many of the alterations that are observed in TBPH loss of function, such as small NMJ with deformed boutons [32], suggesting that altered MT organization could represent at least part of the molecular mechanism behind the NMJ defects observed in TBPH minus flies.

### TDP-43 Activity is Necessary for Microtubule Organization at Presynaptic Terminals

**Figure 3:**
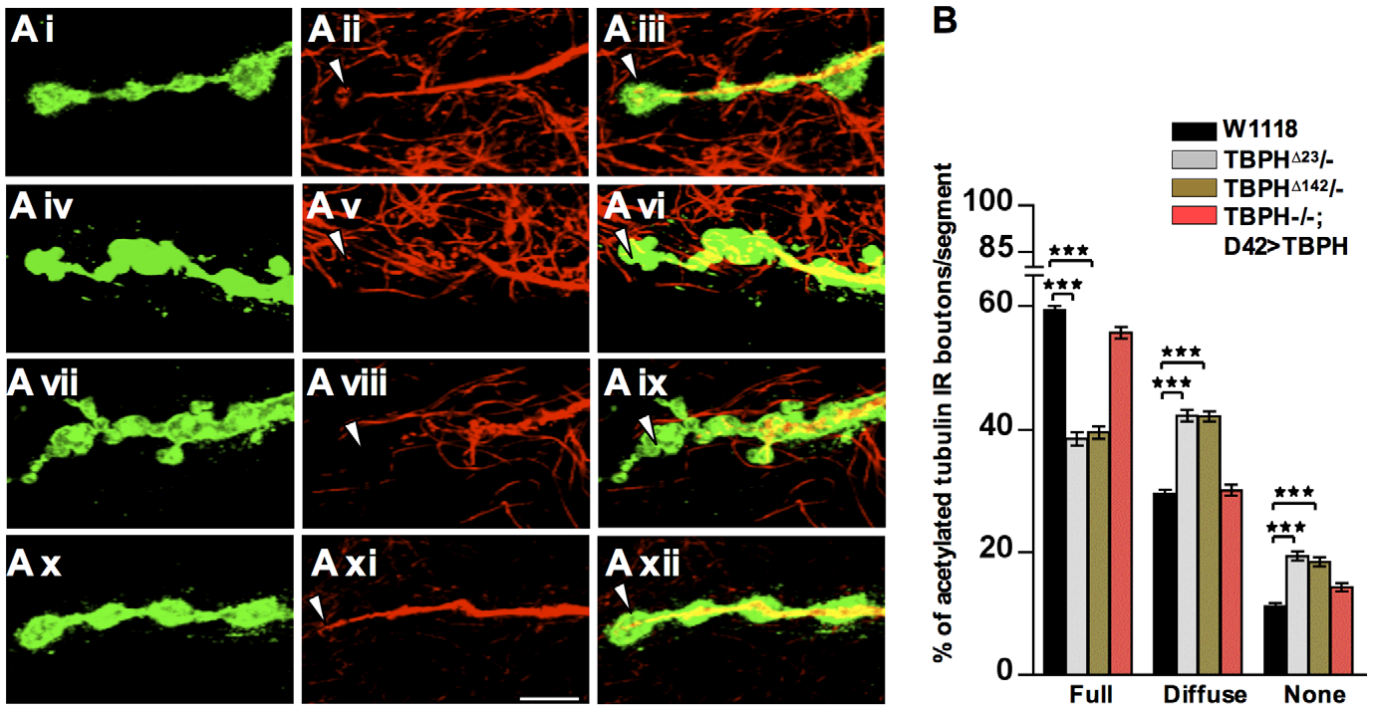
Reduced stable MT and Tubulin acetylation in TBPH mutants. (A) Confocal images showing wild type boutons with stable MT bundles (Ai–Aiii, arrowhead) labeled by acetylated Tubulin. TBPH mutants (Aiv–Avi) TBPH^D23^/-, (Avii–Aix) TBPH^D142^/- show the absence of acetylated MTs staining at the distal boutons (arrowhead). (Ax–Axii) *D42-*GAL4 driven TBPH expression rescues the lack of stable MTs in the TBPH loss of function. Scale bar 5 µm. (B) Quantifications of stable MT labeled by acetylated Tubulin in the abdominal segment II showing significant labeling reduction in TBPH mutant alleles compared to wild type larval NMJ. ***p<0.001 calculated by one-way ANOVA. *n* = 16 larvae.

To test this possibility, we then decided to investigate the MT organization by analyzing *futsch* staining, which labels bundled and unbundled MTs and provides a reliable marker for the cytoskeleton at presynaptic terminals [24], [33], [34]. Anti-*futsch* specific antibody 22c10 was used to stain MTs and big boutons at muscle 6/7 were analyzed. In wild type NMJs, *futsch* labeling highlights bundled MTs and occupies the main part of the presynaptic terminals, filling almost completely the proximal synaptic boutons. In newly formed or distally located synaptic boutons, however, *futsch* staining was fainter, fragmented and more diffuse (Figure 2Ai–Aiii). The *futsch*-staining pattern at each synaptic bouton was scored as percentage of the number of boutons in which *futsch* immunostaining appeared full, diffuse or absent (Figure 2B). Compared with this situation, in TBPH mutant alleles total *futsch* staining was dramatically modified. An increased number of boutons had a diffuse pattern and complete absence of *futsch* was observed at distal terminals (Figure 2Aiv–Avi TBPH^D23^, Avii–Aix TBPH^D142^ and 2B for quantifications). These phenotypes together with *futsch* protein levels, however, could be rescued by expressing the TBPH protein in motoneurons with *D42*-GAL4 (Figure 2Ax–Axii, 2B and 2C) demonstrating that these alterations were specific of TBPH function and suggesting that MT stability in TBPH mutants might be affected in concomitance with *futsch* down regulations. To test this possibility, we performed double staining of presynaptic terminals with antibodies against acetylated tubulin and HRP. Acetylation of specific lysine residue in α-tubulin is a posttranslational modification that marks stabilized MTs and contributes to the regulation of microtubule dynamics [35]. Moreover, increased α-tubulin acetylation and MT stability was noticed in cells transfected with microtubule-associated proteins such as MAP1B, MAP2 or Tau [36]. In TBPH minus alleles, acetylated MTs are significantly reduced in distal boutons (Figure 3Aiv–vi TBPH^D23^, Avii–ix TBPH^D142^ arrow head and 3B for quantifications) compared to prominent tubulin acetylation labeling in wild type terminal boutons (Figure 3Ai–iii and 3B). The decrease in acetylated tubulin labeling was rescued by introducing the endogenous protein back in motoneurons using *D42*-GAL4 (Figure 3Ax–xii and 3B).

### Reduced *futsch* and Microtubules Stability Cause the Presynaptic Defects Observed in TDP-43 Mutants

**Figure 4:**
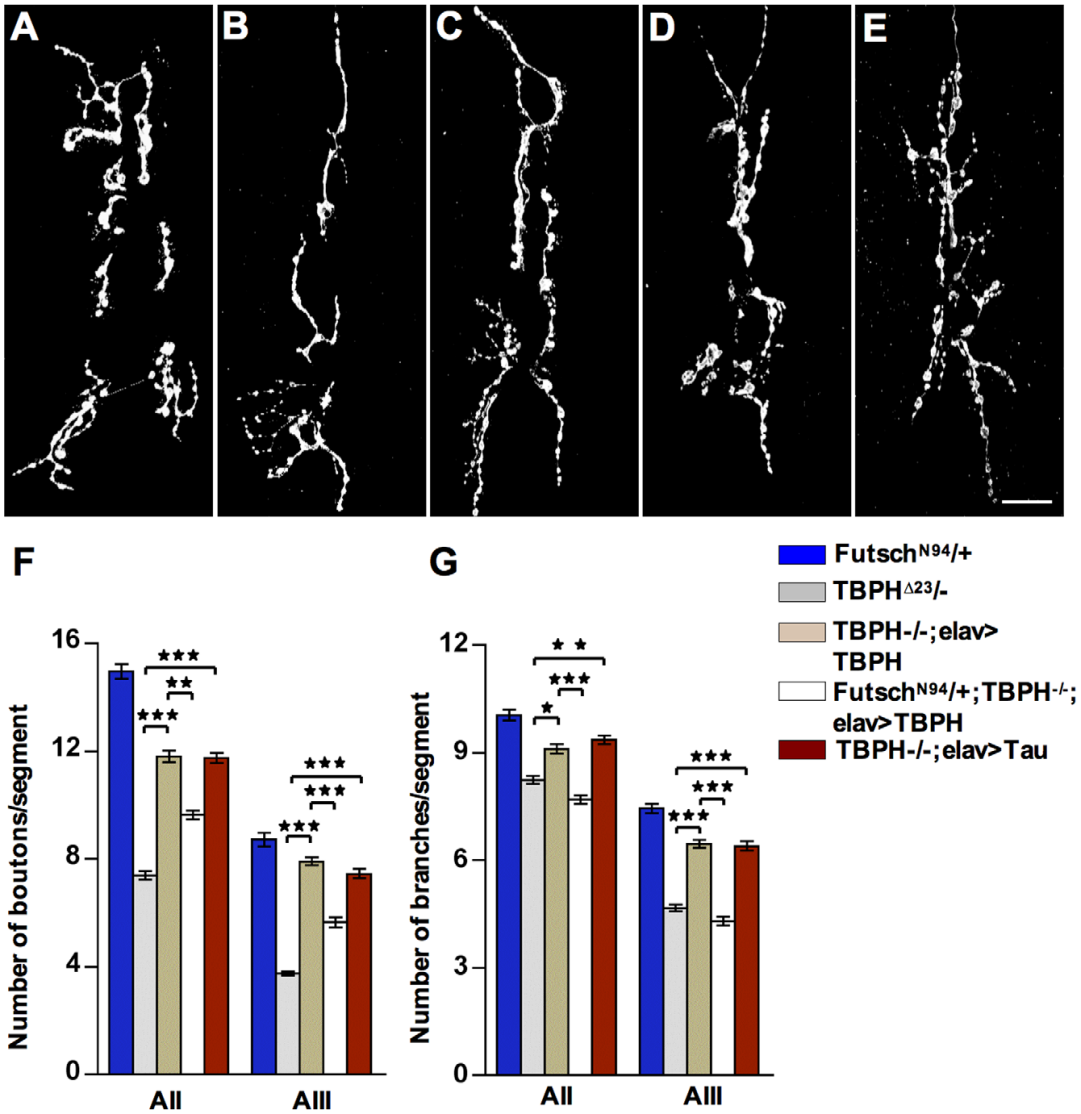
TBPH function requires *futsch* activity to promote NMJ growth. NMJ morphology in muscle 6/7 abdominal segment II of (A) *futsch^N94^*/+ (B) TBPH^D23^/- (C) TBPH^D23^/-;*elav*>TBPH (D) *futsch^N94^*/+;TBPH^D23^/-;*elav*>TBPH (E) TBPH^D23^/-;*elav*>Tau^wt^, Scale bar 20 µm is valid for all figures. Note the NMJ growing defects in *futsch^N94^*/+;TBPH^D23^/-;*elav*>TBPH genotypes compared to TBPH^D23^/-;*elav*>TBPH. Human Tau protein expression showed the recovery in NMJ growing defects observed in TBPH mutants. (F) Quantifications showing significant reduction in the number of boutons and (G) the number of branches in *futsch^N94^*/+;TBPH^D23^/-;*elav*>TBPH genotypes compared to TBPH^D23^/-;*elav*>TBPH. Tau protein expression recovered the NMJ growing defects observed in TBPH mutants. *n* = 13 larvae.

**Figure 5:**
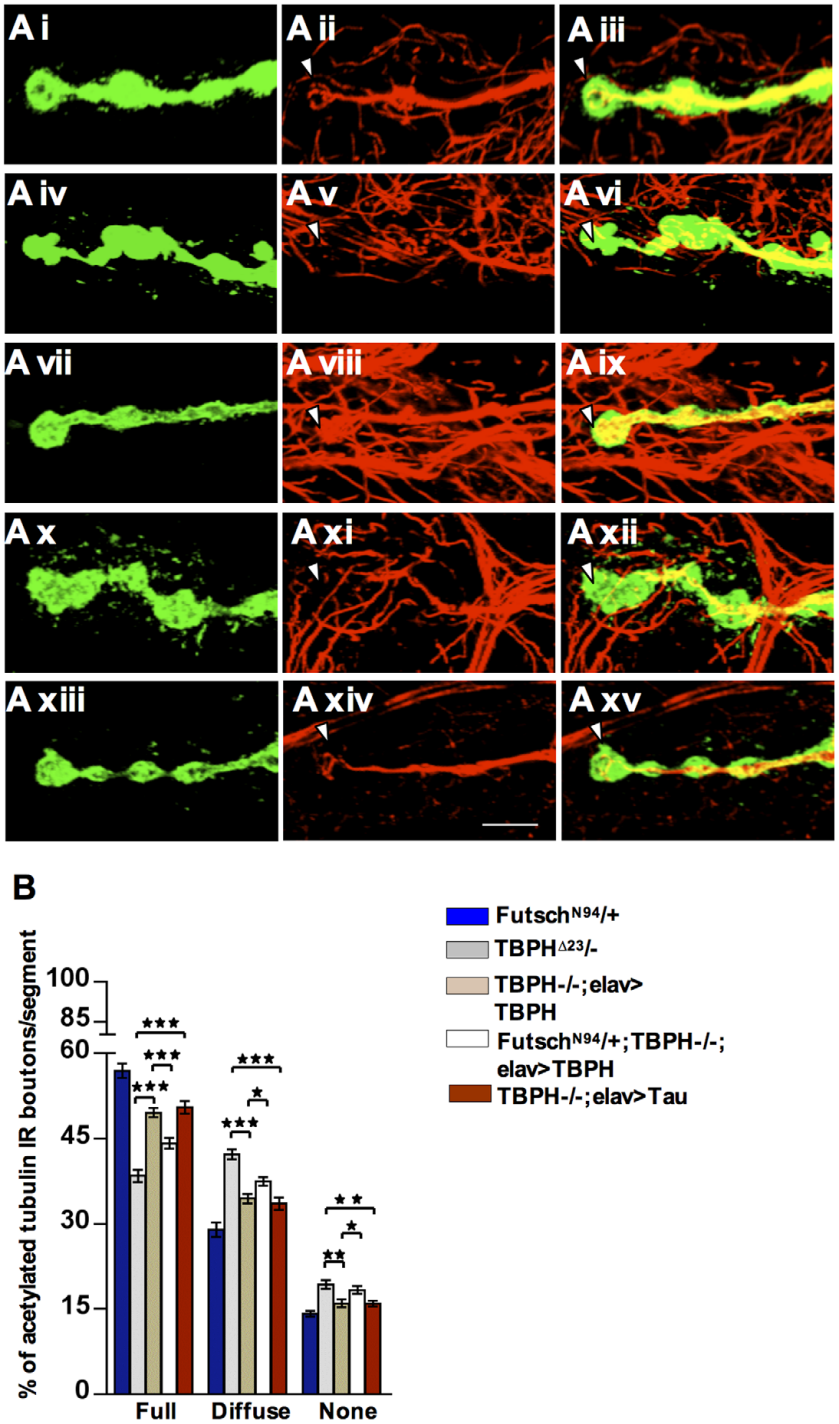
TBPH function requires *futsch* activity to stabilize presynaptic MTs. (A) Confocal images showing the distribution of acetylated MTs in distal boutons of (Ai–Aiii) *futsch^N94^*/+, (Aiv–Avi) TBPH^D23^/-, (Avii–Aix) TBPH^D23^/-;*elav*>TBPH, (Ax–Axii) *futsch^N94^*/+;TBPH^D23^/-;*elav*>TBPH and (Axiii–Axv) TBPH^D23^/-;*elav*>Tau. Note that Tubulin acetylation and stable MT reduction was noticed in the distal boutons of Futsch^N94^/+; TBPH^D23^/-;*elav*>TBPH compared to similar rescue in the TBPH^D23^ mutant background alone (arrow head). Expression of Tau protein in TBPH mutant alleles was able to rescue the distribution of acetylated MTs at synaptic boutons. Scale bar 5 µm valid for all figures. (B) Quantifications of acetylated stable MTs in muscle 6/7 abdominal segments II *n* = 13 larvae. * *p*<0.05, ** *p*<0.01 and ***p<0.001.

Although heterozygous and trans heterozygous combinations between the mutant alleles of *futsch* ^N94^ and TBPH^D23^ did not show major differences in synaptic growth or bouton numbers compared with wild type controls (Figure S4A–B), we observed that the neuronal expression of the TBPH protein in TBPH^D23^ homozygous mutant flies was not able to rescue the presynaptic phenotypes if one copy of *futsch* was removed from the genetic background. Thus, *futsch* ^N94^/+;TBPH^D23^/TBPH^D23^;*elav-*GAL4>TBPH animals (Figure 4D, F–G) compared to +/+; TBPH^D23^/TBPH^D23^; *elav-*GAL4>TBPH flies (Figure 4C, F–G), showed structural defects in the assembled synaptic terminals with reduced number of 1b boutons and terminal branches. At the molecular level, we found that the organization of stable MTs were affected in *futsch* ^N94^/+;TBPH^D23^/TBPH^D23^; *elav-*GAL4>TBPH flies (Figure 5Ax–xii) compared to +/+; TBPH^D23^/TBPH^D23^; *elav-*GAL4>TBPH flies (Figure 5Avii–ix), indicating that TBPH function may requires *futsch* activity to stabilize MTs during synaptic growth and bouton formation. Likewise, we found that stabilization of MTs at motoneurons synaptic terminals was sufficient to rescue the anatomical defects observed in TBPH mutant flies. For these experiments we took the advantage of previous data demonstrating that the neuronal expression of mammalian Tau was capable of rescuing Futsch loss of function phenotypes in *Drosophila* neurons by replacing the microtubule-binding ability of the endogenous protein [37]. Thus, we used a transgenic fly expressing low levels of human Tau in TBPH^D23^ homozygous neurons using *elav*-GAL4. We found that human Tau expression in TBPH^D23^ mutant larvae rescued the reduced number of synaptic boutons and terminal branches in TBPH mutant alleles (Figure 4E, 4F–G). We also found that the rescued boutons were round with a smooth outline and beaded appearance like wild type controls. Furthermore, we observed that the presence of acetylated MTs in distal presynaptic structures was also rescued by human Tau expression (Figure 5Axiii–xv and 5B for quantifications) demonstrating that defects in MT stability were responsible for the morphological defects observed in TBPH mutant boutons. Although, Tau-induced morphological rescue of presynaptic terminals was almost equivalent to the values obtained with the expression of the endogenous TBPH protein itself (Figure 4F–G and Figure 5B) functional recovery of larval motility was not achieved in Tau-rescued flies (Figure S4C) indicating that besides the stabilization of MTs, TBPH may regulates additional functions in motoneurons.

### The TBPH Protein Physically Interacts with *futsch* mRNA

**Figure 6:**
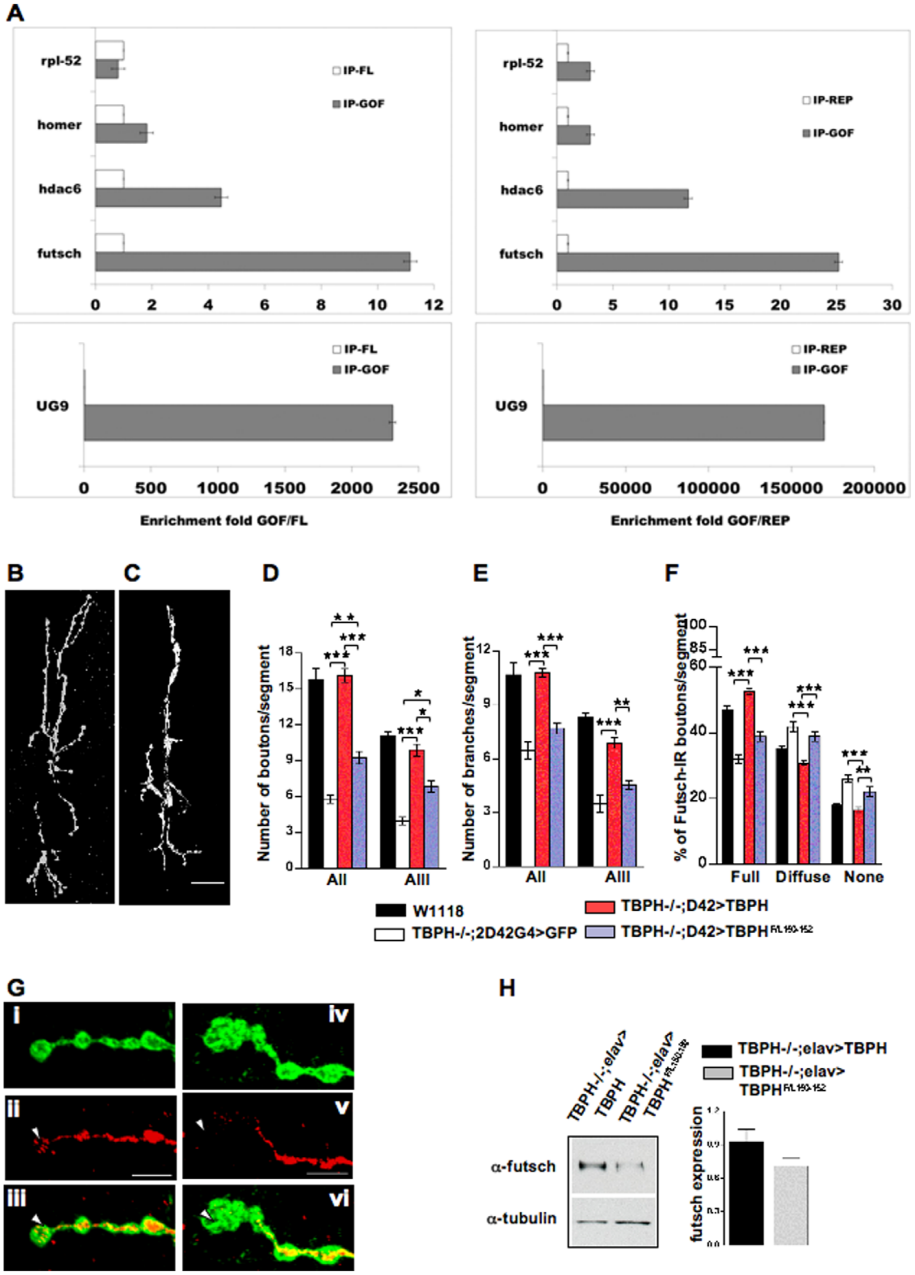
TBPH binding to *futsch* mRNA is required for NMJ growth and MT organization. (A) qPCR analysis of mRNAs immunoprecipitated by Flag-tagged TBPH. The enrichment-fold is referred to an unrelated protein (REP, Right charts) or to the mutant TBPH^F/L150–152^ (FL, Left charts). Significant levels of enrichment were observed for *futsch* mRNA, together with *hdac-6* mRNA and the (UG)9 RNA used as positive controls. The mRNAs of the ribosomal protein *rpl-*52 and homer were not enriched significantively. (B) Wild type TBPH protein expression in TBPH^D23^/-;*D42*>TBPH flies succeed to rescue the synaptic growth compared to (C) TBPH RRM1 mutant isoform in TBPH^D23^/-;*D42*>TBPH^F/L150–152^ larval NMJ, Scale bar 20 µm. (D) Quantifications of big synaptic boutons and (E) terminal branches in muscle 6–7 (abdominal segments II and III) in TBPH^F/L150–152^ and TBPH wild type rescues. (F) Quantifications showing the reduced *futsch* staining in the distal boutons of TBPH^F/L150–152^ rescue NMJs compared to wild type TBPH protein. *n* = 12 larvae. ***p<0.001, **p<0.01. (G) Confocal images showing reduced *futsch* positive staining in the distal boutons of TBPH^F/L150–152^ rescues (Giv-Gvi see arrowhead) compared to wild type TBPH rescues (Gi-Giii). Scale bar 5 µm. (H) Western blot analysis and the respective histogram showing the reduced levels of *futsch* protein expression in TBPH^D23^/-;elav>TBPH^F/L150–152^ compared with TBPH^D23^/-;elav>TBPH (upper panel) *n* = 3. Tubulin was used as a loading control (bottom panel).

Most of the functional properties of TDP-43 described up to now, have been shown to depend very heavily on its RNA binding ability [38]. Therefore, to gain further insights into the mechanisms of TBPH action we first tested for a possible physical interaction between the TBPH protein and *futsch* mRNA (Figure 6). For these experiments we performed immunoprecipitation studies, using a monoclonal anti-Flag antibody, from *Drosophila* heads expressing Flag-tagged TBPH under the control of the *elav*GAL4 promoter (Fig.6A, GOF). Likewise, immunoprecipitation experiments were performed using cells extracts from wild type W1118 flies as well as from flies overexpressing the unrelated Flag-tagged Drosophila protein REEP (Figure 6A, REP) or from flies overexpressing TBPH F/L150–152 (FL), a variant of TBPH mutated within the RNA binding domain (Figure 6A, FL, and see below). Real Time quantitative PCR was used for quantification of the fold-enrichment above controls (GOF/REP and GOF/FL). As expected, the general level of enrichment was higher for GOF/REP with respect to GOF/FL (for example, for futsch we observe approx. 25X enrichment for GOF/REP and 11X for GOF/FL). This is consistent with the fact that the FL mutant differs from the wild-type protein from just two aminoacid substitutions. As a positive control, we verified by RT-qPCR that the protein complex immunoprecipitated by anti-Flag TBPH contained the *hdac-6* mRNA, in agreement with previous publications [39]. On the other hand, we observed negligible levels of enrichments for rpl-52 mRNA, a ribosomal protein with ubiquitous expression (Figure 6A, rpl-52), as well as for homer, a protein enriched in the nervous system (Figure 6A, homer). No enrichment was also observed for rpl-11 mRNA (data not shown). Also as expected, we observed a higher level of enrichment with the REP protein as opposed to the FL mutant when the putative TBPH binding site (UG)9 was added to head extract samples (Figure 6A). Taken together, these data demonstrate that TBPH interaction with the futsch mRNA is highly specific and supports the hypothesis that the observed effects on futsch protein expression might depend on this direct connection.

### The RNA-binding Capacity of TBPH is Essential for its Function *in vivo*

To analyze the functional consequences of this TBPH-*futsch* mRNA physical interaction, we decided to test whether the RNA-binding activity of the protein was responsible for the regulatory roles described above. RNA binding ability of TDP-43 plays an important functional role in alternative splicing and neurodegeneration [40]. In particular, point mutation of two Phenylalanine residues to Leucine (F147L and F149L) in RNP-2 of RRM1 disrupts TDP-43 interaction with RNA, hence describing its importance in RNA recognition [3], [41]. Therefore to check for the importance of this RNA binding activity *in vivo* we generated transgenic flies expressing a mutated form of *Drosophila* TDP-43 in which the conserved Phenylalanine residues present at positions 150 and 152, of RNA recognition motif 1 (RRM-1) in TBPH, were replaced with Leucine. Transgenic flies carrying the TBPH^F/L150–152^ construct under UAS sequences were expressed in neurons using *elav*-GAL4 and it was found that TBPH without RNA binding activity was not able to rescue the TBPH loss of function phenotypes compared with endogenous TBPH, although the intracellular localization of these constructs was similar in both the cases (Figure S5A). Thus we found that expression of TBPH^F/L150–152^ using *D42*-GAL4 in TBPH^D23^ homozygous flies failed to recover the anatomical defects observed in mutant animals regarding the number of synaptic boutons, synaptic branches and MTs organization (Figure 6C–G). Moreover, we found that the biochemical levels of *futsch* protein in TBPH^F/L150–152^ rescue fly heads were not recovered (Figure 6H) although the transgenic expression levels were even higher than the endogenous TBPH protein (Figure S5B) indicating that regulation of *futsch* is specific to the RNA binding capacity of TBPH. Finally, we observed that the functional recovery of fly motility in the form of larval movement (Figure S5C), adult flies walking (Figure S5D) and climbing (Figure S5E) was affected in TBPH^F/L150–152^ rescued flies compared to wild type TBPH expressing controls. Interestingly, however, we did observe that the TBPH^F/L150–152^ construct reached some degree of activity compared to TBPH mutant flies rescued with GFP (Fig. 6D, S5C–E), suggesting that some residual protein activity may exist in the other intact domains. Nonetheless, based on these results we can say that the RNA-binding activity of TBPH, through the RRM-1 domain is definitively essential for its function *in vivo*.

## Discussion

### Evaluating the Potential Functional Connection Between TBPH and *futsch* Expression

Based on the above results, it seems evident that TBPH maintains NMJ growth and MT organization through *futsch* protein action and that TDP-43 RNA binding ability plays a crucial role in this process. Similar to TBPH, other RNA binding protein Fragile X-related (Fxr) gene regulates *futsch* to control synaptic structure and function by directly associating with *futsch* mRNA to alter the expression levels of *futsch* protein. In particular, Fxr acts as a translational repressor of *futsch* to regulate MT dependent synaptic growth and function [34]. Regarding TBPH, therefore, we first of all wanted to determine whether reduced expression of *futsch* protein in our TDP-43 minus flies could be directly related to a reduction in the mRNA levels of these factors. However, quantitative PCR analysis of *futsch* mRNA levels in TDP-43 minus and rescued flies did not show appreciable differences with respect to wild type flies (Fig. S5F). These results suggested that TBPH regulation of *futsch* was not due to differences in RNA stability, transport or translation. In this respect, a visual inspection of the *futsch* gene (FBtr0112628) showed potential UG-repeats in the 5′UTR region near to the ATG codon of the protein that could act as TDP-43 binding site and which could be consistent with a role similar to that of Fxr in affecting mRNA translation. In keeping with this hypothesis, it should be noted that a recent proteomic study performed on the human TDP-43 protein have highlighted its potential interaction with several components of the translational machinery [42], although this has not been confirmed in a subsequent study [43]. Further work, however, will be required to test these hypotheses.

### Concluding remarks

In this work, we show that in our TBPH minus *Drosophila* model the changes observed at the level of NMJs and synaptic boutons formation can be explained by defects at the cytoskeleton level, which in turn are mediated by a down regulation of the *futsch* protein (but not mRNA). These results provide additional insight with regards to potential disease mechanisms mediated by TDP-43 and considerably extend our knowledge with regards to defining the basic molecular functions of this protein. Future work will be aimed at better characterizing more in depth the functional mechanism through which TBPH regulates *futsch* protein levels and how these results can be extended to the human disease model.

## Materials and Methods

### Neuromuscular junctions

To quantify the NMJ growth, embryos from all genotypes were collected during 2 hrs in agar plates and staged at 25°C. Homozygous first, second and third instar larvae were selected against GFP expressing chromosome balancers. Body wall muscles were dissected as per the previous protocols [44]. Primary antibodies α-HRP (Jackson immunoresearch laboratory, 1∶100), α-Synapsin (DSHB, 1∶10), α-Highwire (DSHB, 1∶10), α-Bruchpilot (DSHB, 1∶10), α-DLG (DSHB, 1∶250), α-*futsch* (DSHB, 1∶50), α-tubulin (Calbiochem, 1∶100) and α-acetylated tubulin (Sigma-Aldrich, 1∶2000) and secondary antibodies Alexa 488 Goat-anti rabbit and Alexa 555 Goat-anti mouse IgG (Invitrogen, 1∶500) were used.

**Quantifications of** ***futsch*** **and acetylated Tubulin MT bundles at NMJs:** were done as described in [24], [45] with minor modifications. The bundled appearance of MTs in the proximal boutons in general occupies >75% of the bouton space was considered as full, while boutons in which MTs are fragmented and occupies <75% of bouton space are classified as diffused. Finally the boutons without MT staining are considered as empty boutons.

### Immunostaining

For brains we followed the protocol by Wu et al [46]. Briefly, larval brains were dissected carefully in PB-0.3%Triton-X100 (PBT) buffer and fixed in freshly prepared ice cold 4% paraformaldehyde (PFA) for 30 min. Blocking was done with 5% normal goat serum (NGS) and primary antibodies α-Flag (Sigma, 1∶200) and α-elav (DSHB, 1∶250) was used. Samples were incubated at 4°C overnight and treated with fluorescent conjugated secondary antibodies Alexa 488 Goat-anti rabbit IgG and Alexa 555 Goat-anti rat IgG (Invitrogen, 1∶500) for 2 hrs at room temperature. All primary and secondary antibodies were diluted in PBT-5% NGS. Slow fade gold antifade reagent (Invitrogen) was used as a mounting medium and the samples were analyzed with a confocal laser-scanning microscope.

### Larval movement

To evaluate the peristaltic waves of third instar larvae, we followed our previously established protocol [8]. Briefly, Individual larvae were selected and washed carefully with water to remove any remaining food attached to the body. The larvae were carefully transferred with the help of forceps to 0.7% agarose plates (100 mm). Under the stereoscope, larvae were allowed to adopt for 30 sec and start counting the peristaltic waves per 2 min. Minimum 25 larvae per each genotype were counted individually and average has been taken.

### Walking Assay

Walking ability of young flies with the age of 3–4 days was performed as described earlier [8]. Briefly, individual fly was placed on transparent 145 mm diameter petri plate whose bottom was marked with 1×1 cm square grid lines. Flies were allowed to adopt for at 30 sec and walking was analyzed by counting the number of 1×1 grids crossed by the fly. Minimum 50 flies were tested individually from each genotype and the average has been taken.

### Climbing assay

Climbing ability of different genotypes was performed in an empty transparent Duran 50 ml glass cylinder. The cylinder was divided into three parts as bottom, middle and top. Age matched flies to be tested, were dropped at the bottom of the cylinder carefully and climbing ability was scored based on the flies that are moving onto the top in 15 sec. Three trials was performed on each day and the average was taken as climbing ability. Thirty flies per batch and minimum of 100 flies in each genotype were tested.

### Bouton shape

Boutons with round and smooth outline having equal diameter on both the axis were considered as regular boutons, whereas the boutons with deformed shape having rough outline and fusiform appearance are treated as irregular boutons.

### Fly stocks

W1118, OregonR, *Futsch* ^N94^, *D42*-GAL4, *elav*-GAL4, UAS-CD8-GFP were obtained from Bloomington Indiana. UAS-Tau^wt^ was gifted by Mel Feany.

### Western blot analysis

Protein was extracted using lysis buffer containing 10 mM Tris HCl, pH-7.4, 150 mM NaCl, 5 mM EDTA, 5 mM EGTA, 10% Glycerol, 50 mM NaF, 5 mM DTT, 4 M Urea and protease inhibitors (Complete mini EDTA free from ROCHE). The extracted protein was separated in SDS polyacrylamide gels and blotted on to 0.2 µm nitrocellulose membranes (Sigma-Aldrich). Membranes were blocked and incubated with primary antibodies overnight. Primary antibodies such as α-TBPH (1∶3000), α-Wit-C (DSHB, 1∶200), α-FasII (DSHB, 1∶ 100), α-Spectrin (DSHB, 1∶3000), α-Bruchpilot (DSHB, 1∶300), α-Tubulin (Calbiochem, 1∶3000) and α-Actin (Sigma A2066) were used. Goat anti-rabbit/anti-mouse igG HRP conjugated were used as secondary antibodies (1∶100000, Pierce). Proteins detection was done with Femto Super Signal substrate (Pierce, 1∶10).

### Western blots for *futsch* protein levels

Western blot analysis to detect *futsch* protein was done in agreement with Zou. *et al* [47]. Briefly, 20 fly heads were homogenized in ice cold lysis buffer containing 1% CHAPS, 20 mM Tris/HCl (pH 7.5), 10 mM EDTA, 120 mM NaCl, 50 mM KCl, 2 mM DTT and protease inhibitors (Roche, Complete Mini EDTA free). The homogenization step was followed by incubation in ice and centrifugation at 9000 *g* for 10 min at 4°C. Supernatants were collected and approximately 30 µg of total protein was loaded in pre-cast gradient gels with NuPAGE (Invitrogen NuPAGE® Novex 3–8% Tris-Acetate Gel 1.0 mm, NuPAGE LDS Sample buffer, NuPAGE reducing agent). The upper part of the gel, up to molecular weight 250 kDa, were placed on filters soaked with 1% SDS and transferred to nitrocellulose membrane at 0.12 amp current for 16 hrs in 20 mM Tris and 150 mM Glycine [31]. The lower part of the gel, for tubulin as a loading control, was transferred to nitrocellulose in 20% methanol, 20 mM Tris and 150 mM Glycine for 1 hr, at 350 mA current. Blocking with 5% milk in TBS-T 0.1% Tween20 followed by incubation with primary antibody α-*futsch* (DSHB, 1∶400) and α-tubulin (Calbiochem, 1∶3000). After extensive washes, membranes were incubated with secondary antibody anti mouse HRP-labeled (Pierce), diluted 1∶100000 and developed in Femto Super Signal substrate (Pierce, 1∶10).

### Quantitative Real-time PCR analysis

Total RNA was extracted from the heads of wild type, TBPH minus alleles and the endogenous rescues (using D42-Gal4 and elav-Gal4) by using Trizol reagent (Invitrogen) as per the manufacturer's protocol. cDNA was synthesized with 1 µg of RNA sample by using M-MLV Reverse transcriptase (Invitrogen) and exameric random primers. Specific primers were designed to amplify *futsch* gene (*futsch*_254s, TTTCGGGATCCAACGGCTTTAAC; *futsch*_349as (96bp), GCGTCCAGTCGGTCTAGG and the gene expression levels was checked by real-time PCR using SYBR green technology. House keeping gene Rpl-11 (Forward, CCATCGGTATCTATGGTCTGGA and reverse, CATCGTATTTCTGCTGGAACCA) was amplified and used to normalize the results. All amplifications were performed on CFX96™, Real-time PCR detection system (Bio-Rad). The relative expression levels were calculated according to the following equation: ΔC_T_ = C_T(Target)_-C_T(normalized)_.

### Immunoprecipitation and RNA identification by RT-PCR

Protein G magnetic beads (Invitrogen) were washed two times with PBS+0.02% tween and coated with anti-FLAG M2 monoclonal antibody (Sigma). Fly heads (W1118, elav>TBPH, elav>FL and elav>REP) were homogenized in a lysis buffer containing 20 mM Hepes, 150 mM NaCl, 0.5 mM EDTA, 10% Glycerol, 0.1% Triton X-100, and 1 mM DTT with a Dounce homogenizer, followed by centrifugation for 5 min at 0.4 g. RNA containing [19]9 repeats, prepared as described by Buratti and collaborators [3], was added to the head extracts in order to control the efficiency of selection between non-specific and specific purification. The pretreated beads and head extracts were mixed and incubated for 30 min at 4°C, followed by washing five times with lysis buffer. Bound RNA transcripts were extracted with DynaMagTM-Spin (Invitrogen). The beads were treated with Trizol (Ambion) and precipitated with glycogen and Isopropanol. First-strand synsthesis was achieved with Superscript™-III (Invitrogen). Real Time PCRs were carried out with gene specific primers. The used primers are the following:**.** ***Futsch:*** 5′*-*CTCGCCAAAGCCCACATCACC-3′ and 5′-GTCACCCTCACACTCAGCTCC-3′. **homer:** 5′-GGTATAAACTGCTGCGGAAG-3′, and 5′-GACACTGATGATGCGGTAC-3′. ***hdac-6:*** 5′-CGAGCGGCTGAAGGAGAC-3′ and 5′-ACCAGATGGTCCACCAATTCG-3′. ***rpl-52:*** 5′-GAAAATAACAAAGATCTGCTTGGCC-3′ and 5′-AAGTGGCCCTTGGGCTTCAG-3′. Specific reverse transcription of [19]9 RNA was carried out with pBSKS 929_950as oligo 5′-AGCGGGCAGTGAGCGCAACGCA-3′. Amplification of [19]9 transcripts was obtained with the following oligos: pBSKS 667_687s 5′-TGGCGGCCGCTCTAGAACTA-3′ and pBSKS 903_924s 5′-ATGTGAGTTAGCTCACTCATTA-3′.

In order to calculate the enrichment fold, initially, all data were normalized to the respective inputs. Then, the signal was represented by how many more fold increase was measured compared to the control signal. To this aim, the enrichment was calculated by subtraction of controls ΔCt (unrelated REP protein (IP-REP) or TBPH^F/L150-152^ (IP-FL), a variant of TBPH mutated within the RNA binding domain control) from ΔCt of experimental sample overexpressing TBPH-wt (IP-GOF).

The results were derived from three independent immunoprecipitation experiments and error bars represent standard deviations on the normalized ratios.

The statistical significance of differences observed between control- and specific- immunoprecipitation samples was determined by t-test (p<0.05).

### Data analysis and statistics

Total number of boutons and branches were acquired from longitudinal muscle 6/7 of hemisegments A2 and A3 of all genotypes. Branches were defined as an extension of the presynaptic motor neuron that has not less than two or three boutons. Immunoreactivity of *futsch* and acetylated tubulin was quantified from the images acquired at the same fluorescence intensity using Zeiss LSM510 confocal microscope. Statistics were performed using GraphPad Prism version 4.0b software. One-way ANOVA was performed using Bonferroni's multiple comparison test to compare two or more independent groups. The significance between the variables was showed based on the p-value obtained (*indicates p<0.05, ** p<0.01 and *** p<0.001). All the numbers in the histograms represent mean ± SEM.

## Supporting Information

Figure S1 **Loss of TBPH does not affect muscles development or synaptic stability.** (A, D) Wild type body wall muscles stained with phalloidin and tubulin respectively. Similar stainings in (B, E) TBPH^D23^/-, (C, F) TBPH^D142^/- showed no changes in muscle morphology and cytoskeleton organization. (G) Quantifications showing no significant difference between the wild type and the TBPH minus muscles during larval development. (H) Confocal images of postsynaptic DLG protein showing no pre-synaptic retractions in (Hi–Hiii) wild type, (Hiv–Hvi) TBPH^D23^/- and (Hvii–Hix) TBPH^D142^/- larval NMJ. Scale 5 µm. (I) Percentage of third instar larvae NMJs presenting footprints showed no significant differences between wild type and TBPH minus alleles. The numbers of NMJs analyzed per each genotype is indicated above the columns.
(TIF)

Figure S2 **TBPH loss of function does not affect motoneurons formation and survival**. (A) *D42-*GAL4 driven expression of GFP protein in dorsal medial clusters of motoneurons in (Ai–Aiii) wild type background and in (Aiv–Avi) TBPH^D23^/- background labeled similar cellular populations. Scale bar 20 µm. (B) Quantification of the number of GFP positive motoneurons present in the dorsal medial cluster of different abdominal segments at the ventral ganglion. No differences between wild type flies and TBPH mutant alleles were observed. *n* = 7 larvae.
(TIF)

Figure S3 **Subcellular localization patterns and protein expression levels of different presynaptic proteins involved in NMJs formation were not affected by TBPH depletion.** (A) Confolcal images showing the distribution of the active zone marker Bruchpilot in (Ai–Aiii) wild type, (Aiv–Avi) in TBPH^D23^/- and (Avii–Aix) in TBPH^D142^/-. Other presynaptic terminal markers such as (Bi-Bix) Synapsin showed no difference in their localization in TBPH null alleles compared to wild type. Scale 5 µm. Western blots analysis showing no difference in the expression levels of pre-synaptic proteins (C) Fas-II, (D) Wit-C, (E) Bruchpilot and (F) Spectrin. Note that tubulin was used as a loading control in the bottom panel of each blot and its expression levels were further corroborated against actin used as a second loading control (G).
(TIF)

Figure S4 **Genetic interactions between TBPH and** ***futsch*** **transheterozygous flies.** Quantitative analysis of (A) number of big synaptic boutons and (B) terminal branches in muscle number 6/7 abdominal segments II and III. Heterozygous and trans heterozygous alleles of *futsch^N9^* ^4^ and TBPH^D23^ present no significant changes in the number of big synaptic boutons and synaptic branches compared to wild type larval NMJ. (C) Larval motility in the third instar larvae showing no rescue with the Tau protein expression in TBPH mutant background compared to similar rescue with endogenous TBPH expression. (*n* = 40 larvae for each genotype).
(TIF)

Figure S5 **Functional comparisons between TBPH wild type protein and the RNA binding defective TBPH^F/L150-152^ isoform (**A) Nuclear localization of TBPH wild type protein (Ai–Aiii) and TBPH ^F/L150–152^ (Aiv-Avi) in the neuronal cell bodies of the dorsal medial motor neurons of ventral ganglion. Scale bar 20 µm. (B) Expression levels of TBPH full-length protein and TBPH^ F/L150–152^ in the fly heads expressed with *elav*-Gal4 (upper panel). Tubulin was used as a loading control (bottom panel). (C) TBPH^F/L150–152^ rescues the motility defects such as larval movement, (D) defects in walking (*n* = 50) and (E) climbing (*n* = 250). (F) Real-time PCR quantifications of the *futsch* transcript levels in the heads from wild type, TBPH minus alleles and endogenous TBPH rescue with *D42-*Gal4 and *elav-*Gal4. Four independent experiments were quantified and the average is plotted in the graph.
(TIF)
