# Supplementary figures and images for: TDP-43 Regulates Drosophila Neuromuscular Junctions Growth by Modulating Futsch/MAP1B Levels and Synaptic Microtubules Organization
Source: PLoS One. 2011 Mar 11;6(3):e17808. doi: 10.1371/journal.pone.0017808 (PMC3055892; doi:10.1371/journal.pone.0017808)

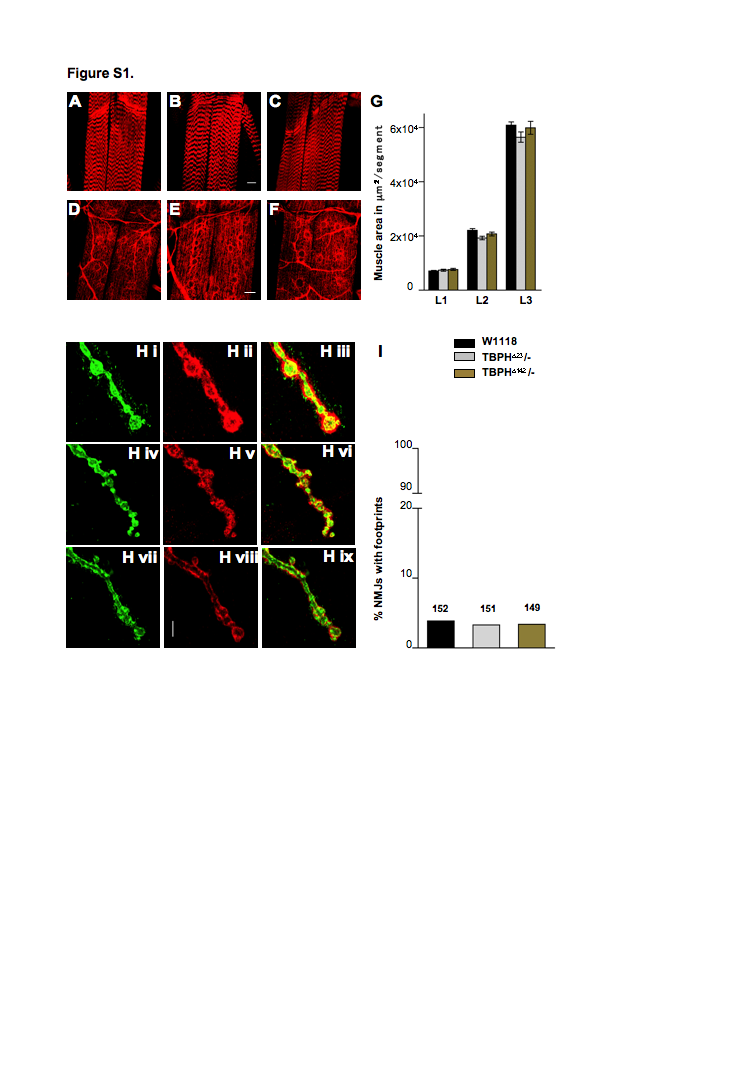

Supplement: Figure S1 — Loss of TBPH does not affect muscles development or synaptic stability. (A, D) Wild type body wall muscles stained with phalloidin and tubulin respectively. Similar stainings in (B, E) TBPHD23/-, (C, F) TBPHD142/- showed no changes in muscle morphology and cytoskeleton organization. (G) Quantifications showing no significant difference between the wild type and the TBPH minus muscles during larval development. (H) Confocal images of postsynaptic DLG protein showing no pre-synaptic retractions in (Hi–Hiii) wild type, (Hiv–Hvi) TBPHD23/- and (Hvii–Hix) TBPHD142/- larval NMJ. Scale 5 µm. (I) Percentage of third instar larvae NMJs presenting footprints showed no significant differences between wild type and TBPH minus alleles. The numbers of NMJs analyzed per each genotype is indicated above the columns. (TIF) [file pone.0017808.s001.tif]

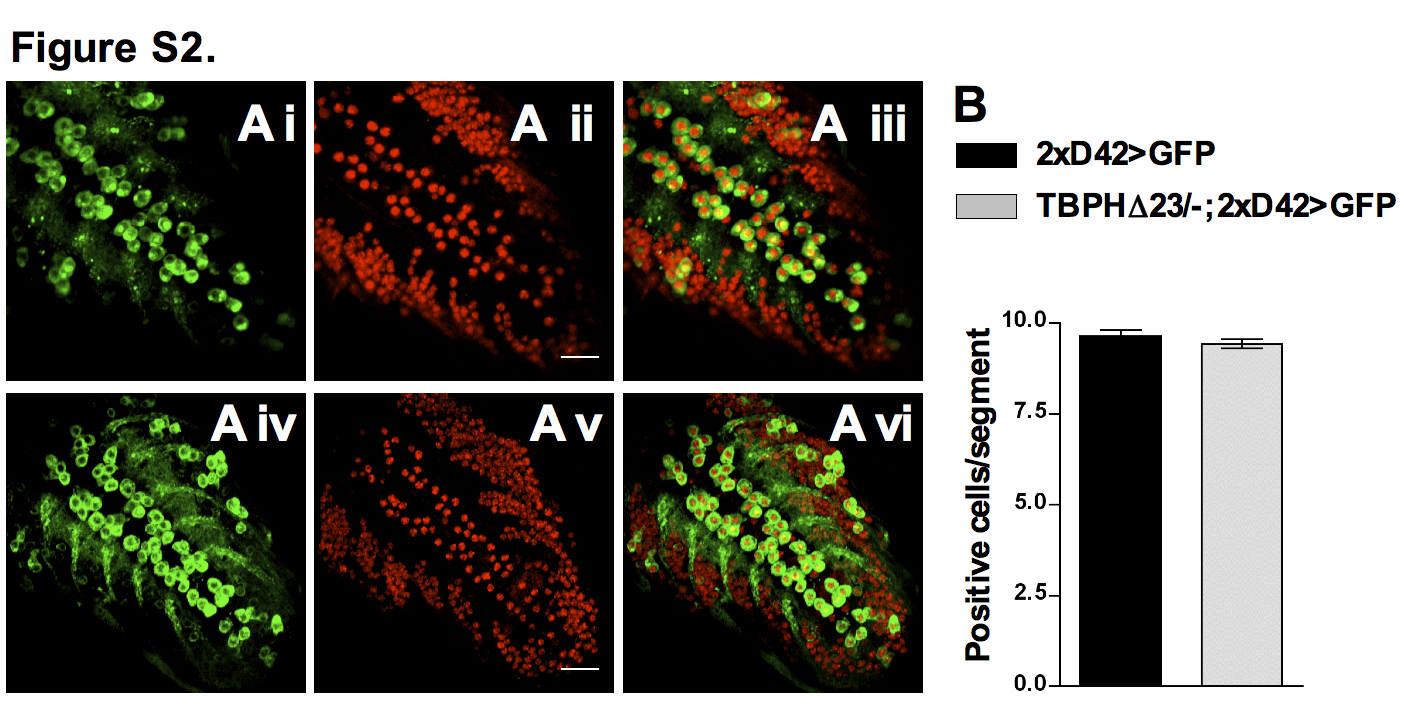

Supplement: Figure S2 — TBPH loss of function does not affect motoneurons formation and survival. (A) D42-GAL4 driven expression of GFP protein in dorsal medial clusters of motoneurons in (Ai–Aiii) wild type background and in (Aiv–Avi) TBPHD23/- background labeled similar cellular populations. Scale bar 20 µm. (B) Quantification of the number of GFP positive motoneurons present in the dorsal medial cluster of different abdominal segments at the ventral ganglion. No differences between wild type flies and TBPH mutant alleles were observed. n = 7 larvae. (TIF) [file pone.0017808.s002.tif]

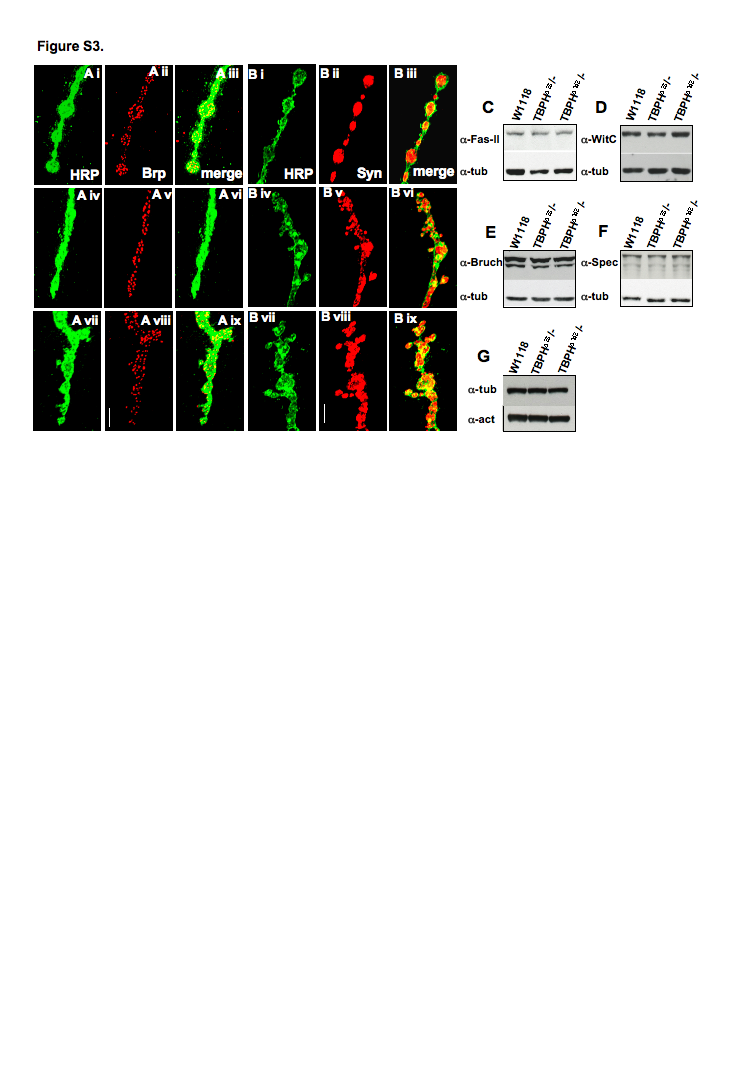

Supplement: Figure S3 — Subcellular localization patterns and protein expression levels of different presynaptic proteins involved in NMJs formation were not affected by TBPH depletion. (A) Confolcal images showing the distribution of the active zone marker Bruchpilot in (Ai–Aiii) wild type, (Aiv–Avi) in TBPHD23/- and (Avii–Aix) in TBPHD142/-. Other presynaptic terminal markers such as (Bi-Bix) Synapsin showed no difference in their localization in TBPH null alleles compared to wild type. Scale 5 µm. Western blots analysis showing no difference in the expression levels of pre-synaptic proteins (C) Fas-II, (D) Wit-C, (E) Bruchpilot and (F) Spectrin. Note that tubulin was used as a loading control in the bottom panel of each blot and its expression levels were further corroborated against actin used as a second loading control (G). (TIF) [file pone.0017808.s003.tif]

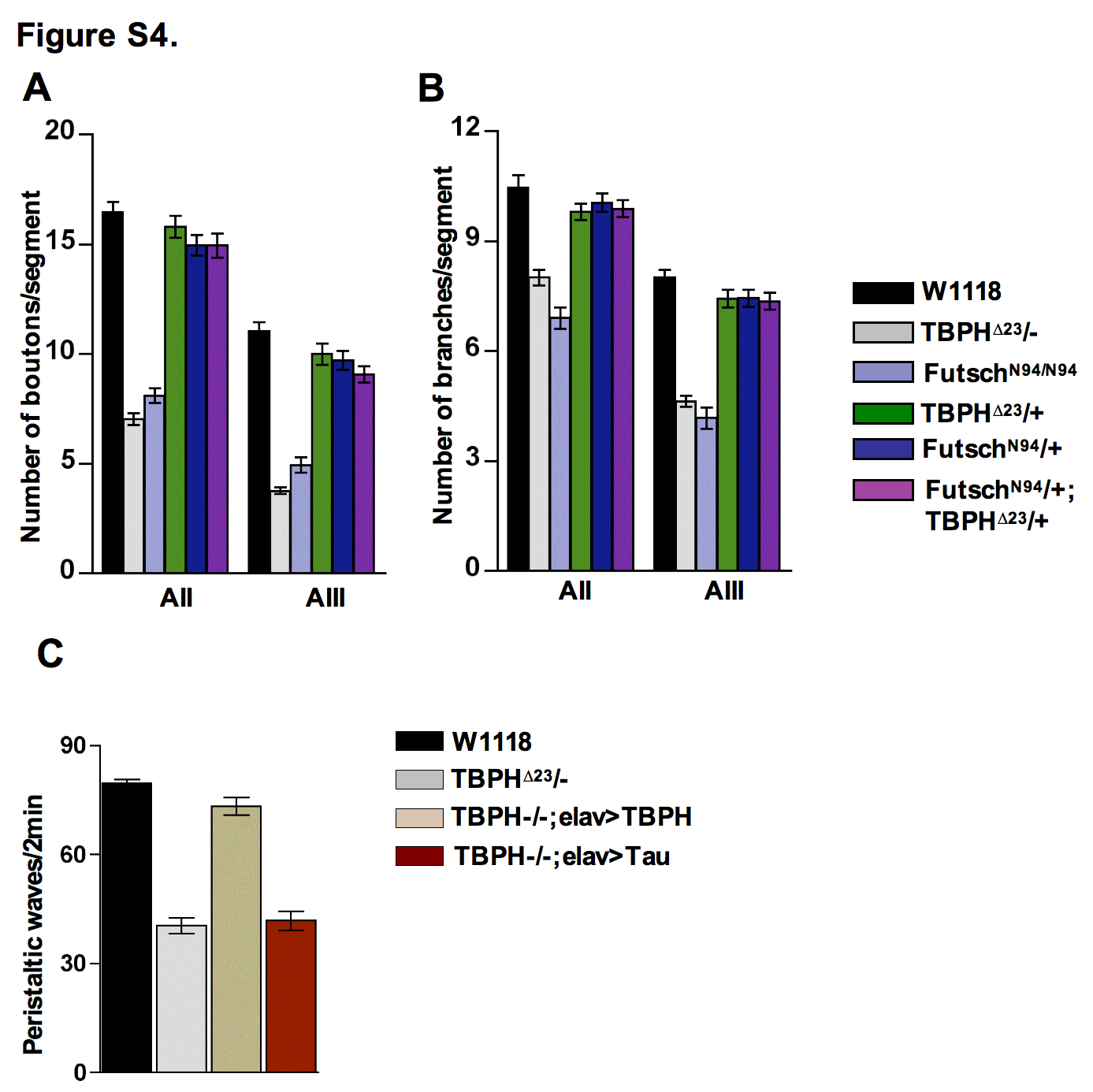

Supplement: Figure S4 — Genetic interactions between TBPH and futsch transheterozygous flies. Quantitative analysis of (A) number of big synaptic boutons and (B) terminal branches in muscle number 6/7 abdominal segments II and III. Heterozygous and trans heterozygous alleles of futschN9 4 and TBPHD23 present no significant changes in the number of big synaptic boutons and synaptic branches compared to wild type larval NMJ. (C) Larval motility in the third instar larvae showing no rescue with the Tau protein expression in TBPH mutant background compared to similar rescue with endogenous TBPH expression. (n = 40 larvae for each genotype). (TIF) [file pone.0017808.s004.tif]

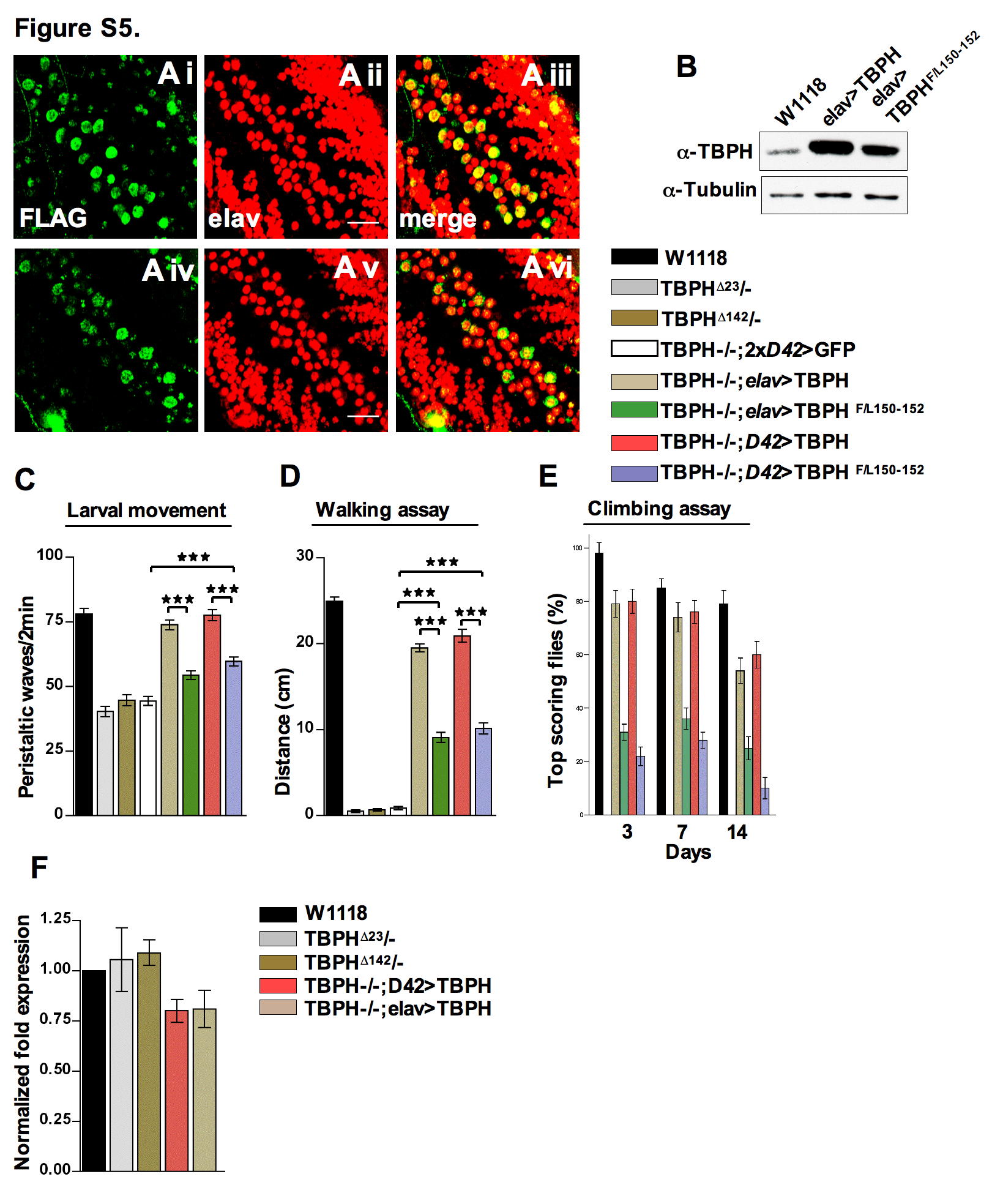

Supplement: Figure S5 — Functional comparisons between TBPH wild type protein and the RNA binding defective TBPHF/L150-152 isoform (A) Nuclear localization of TBPH wild type protein (Ai–Aiii) and TBPH F/L150–152 (Aiv-Avi) in the neuronal cell bodies of the dorsal medial motor neurons of ventral ganglion. Scale bar 20 µm. (B) Expression levels of TBPH full-length protein and TBPH F/L150–152 in the fly heads expressed with elav-Gal4 (upper panel). Tubulin was used as a loading control (bottom panel). (C) TBPHF/L150–152 rescues the motility defects such as larval movement, (D) defects in walking (n = 50) and (E) climbing (n = 250). (F) Real-time PCR quantifications of the futsch transcript levels in the heads from wild type, TBPH minus alleles and endogenous TBPH rescue with D42-Gal4 and elav-Gal4. Four independent experiments were quantified and the average is plotted in the graph. (TIF) [file pone.0017808.s005.tif]
